# Brazilian Food Banks: Overview and Perspectives

**DOI:** 10.3390/ijerph182312598

**Published:** 2021-11-29

**Authors:** Natalia Tenuta, Thaís Barros, Romero Alves Teixeira, Rômulo Paes-Sousa

**Affiliations:** 1 René Rachou Institute, Oswaldo Cruz Foundation (IRR-Fiocruz), Belo Horizonte 30190-002, Brazil; romulo.paes@fiocruz.br; 2 Department of Nutrition, Federal University of Vales do Jequitinhonha e Mucuri, Diamantina 39100-000, Brazil; thais.barroscvo@gmail.com (T.B.); romero.teixeira@ufvjm.edu.br (R.A.T.)

**Keywords:** food banks, food losses and waste, food security, food and nutrition education, Brazil

## Abstract

In recent decades food banks have become a worldwide response to the contradicting the coexistence of food losses and waste, on the one hand, and hunger and food insecurity on the other. In Brazil, food banks had a rapid expansion, becoming the object of public policy on Food and Nutrition Security and of non-profit private institutions. Our study presents an unprecedented overview of all the food banks currently active in the Brazilian territory, discussing their performances and perspectives. We conducted descriptive research, aiming to characterize the number, spatial distribution, performance, and modalities of operation of the Brazilian food banks. We mapped 217 active food banks and they all participated in the study. The results revealed the important capillarity of the food banks, which exist in all 27 Brazilian federative units, but also demonstrate the potential and need for expansion. Most of the Brazilian food banks has commercial establishments as their largest donor partners and have fruits and vegetables as their most donated items. They mostly complement the feeding of families at social risk and children served by social institutions. Food and nutrition education actions are offered by all the studied units to donor partners and beneficiary institutions and families.

## 1. Introduction

Over the past six decades, food banks have gained worldwide expression as an important and strategic response to the contradictory scenario in which food losses and waste (FLW) and food insecurity coexist. Although co-opted, in some regions of the world, by an uncritical logic based on charity [1], in other places, including Brazil, food banks are recognized for their positive impacts in reducing FLW and have been contributing to minimize the hunger and food insecurity caused by social inequalities [2,3].

The Food and Agriculture Organization of the United Nations (FAO) [4] states that, worldwide, about one-third of the food produced for human consumption is lost or wasted each year, which is equivalent, on average, to 1.3 billion tons, representing an annual economic loss of USD 940 billion, and emission of 4.4 gigatons of greenhouse gases. Food losses are defined as resulting from inadequate or inefficient procedures that cause loss or damage to food products in the processes of handling, transformation, storage, transportation, and packaging [5]. Food waste, on the other hand, refers to the reduction in the volume of food suitable for human consumption that occurs in the final stage of the food chain. In other words, it is a phenomenon associated with the inefficiency of the distribution (both wholesale and retail) and consumption processes and has a close relationship with conscious food consumption [6].

Food is a right for everyone and must be guaranteed, through actions of food and nutritional security, so that “all people, at all times, have physical and economic access to sufficient, safe and nutritious food that meets their dietary needs and food preferences for an active and healthy life” [7]. However, according to the report “The State of Food Security and Nutrition in the World” [8], in 2018 more than 820 million people (10.8%) in the world were in a state of malnutrition. Based on this contradiction, food insecurity can be defined as a situation “when people do not have adequate physical, social, or economic access to food” [9].

In this scenario, food banks play a strategic and responsible role by capturing food that is about to be lost or wasted and reintroducing it to the supply chain, providing access to adequate and safe food for people in situations of social, food, and nutritional vulnerability.

The first food bank was created in 1966 in the United States, and its original idea was to connect surplus food, with no commercial value, to the needs of a vulnerable population [1,10]. The food bank proposal expanded, reaching Canada in 1981, the United Kingdom in 1986, Spain in 1987, and Brazil in 1994, until it gained a global scale [11]. Presently, there are food banks in operation on all continents, many of them organized and articulated in major international, regional, and national networks. Currently, the Global Food Banking Network is the largest network which connects and empowers food banks affiliated to regional and national food banks networks in more than 40 countries [12].

The food banks operating around the world are generally initiatives of civil society that has a common modus operandi by collecting, storing, and distributing food donations of no commercial value to social welfare institutions or directly to vulnerable families. However, some of them have specific characteristics: in some countries, such as Canada, the government facilitates and encourages food donations; in the United States and Europe, the government provides financial resources to purchase food that complements donations [9,13,14,15,16].

The development of food banks in Brazil was late compared with some other countries, and it was marked by several peculiarities regarding those responsible for the initiatives, the operating ways, financiers, and supporters. The first Brazilian food bank (called Mesa São Paulo) was created in 1994 by private initiative of the Social Service of Commerce (Sesc) in São Paulo, the biggest city of the country, and mostly mirrored the United States model [17,18].

In the following year, the non-governmental organization ONG Banco de Alimentos was implemented as the first initiative mobilized by the civil society, also in São Paulo [17,18]. Later, in 2000, Mesa São Paulo became the Mesa Brasil Sesc Program and gained national status, expanding the initiative to other Brazilian regions. It currently presents active food banks in all 27 Brazilian federative units, including the federal district [19].

At the governmental level, a municipal food bank installed in Santo André (SP) in 2004 was the first experience of public initiative and management [17]. In the same year, a collective of private companies created a food bank in the state of Rio Grande do Sul. In three years, other units were implemented throughout that state, starting the first Brazilian food bank network, the Rede de Bancos de Alimentos do Rio Grande do Sul, in 2010 [20].

All the Brazilian food bank initiatives presented above were initially mirrored in models from other countries, and most of them were driven by mobilization of the civil society. However, by their potential, food banks were incorporated in 2003 as an object of public policies in the Brazilian federal government’s Fome Zero Strategy, integrating the axis of actions articulating the access to food [21]. Through the National Department for Food and Nutritional Security (SESAN) of the now extinct Ministry of Social Development and Fight against Hunger (MDS), the Brazilian government supported the implementation of food banks in states, municipalities, and supply centers. In this program, was financed the preparation of basic architectural and engineering projects, the construction of the facilities, and the acquisition of equipment and consumables, expanding the fight against food losses and waste in the urban and peri-urban agrifood chain throughout the country [22,23].

One of the guidelines of Brazil’s National Food and Nutritional Security Policy (PNSAN) indicated the need to “promote universal access to adequate and healthy food and supply and structuring of sustainable and decentralized systems”. In consonance with this guideline, in 2003 the Food Banks Program was created and supported by the Brazilian federal government. This important public program became a strategic public facility to complement food and promote the human right to adequate food in the country [24].

The creation and expansion of Brazilian food banks took place through different routes and incentives. However, as well as the facilities in other countries, the experiences in Brazil have common characteristics concerning their fundamental goals and the ways of operating, which are reflected in the most recent definition of food banks provided by Ordinance No. 17, of 14 April 2016, which established the Brazilian Food Bank Network: “Food banks are physical and/or logistical structures that offer the service of collecting and/or receiving food donated by private and/or public sectors and freely distributing it to public or private institutions characterized as providers of social assistance services, protection and civil defense, teaching and justice units, health establishments, and other food and nutrition units” [25].

In this operational format, food banks search for potential food loss or waste along the food production and supply chain and, after receiving donations, the collected food goes through selection, classification, sanitation, portioning, and packaging. It is then distributed to social institutions, complementing the food of the beneficiary audience with products in safe conditions for consumption and with guaranteed nutritional quality [2].

Brazilian food banks are guided by three fundamental goals: (i) the combat of food losses and waste, by reintroducing food that was about to be lost or wasted into the supply chain; (ii) the guarantee of food and nutritional security, contributing to the human right to adequate and healthy food of the vulnerable beneficiary population; and (iii) the performance of food and nutrition education actions, aiming at qualifying the agenda to promote adequate and healthy food [2]. This guiding tripod allows to integrate the efforts to improve the conditions of food, nutrition, and health of the Brazilian population.

Technical and scientific investigations and evaluations are basic and systematic elements for decision-making in the scope of any strategy, policy, or social program. These studies are fundamental to gather evidence that supports responses to qualify operational and management performance and contribute more effectively to overcoming or mitigating a social problem [26]. Food banks are the subject of scientific investigations and technical-operational assessments in the international literature, raising thought-provoking questions about the fundamental goals, the design, and logistics of such facilities. In Brazil, audits and technical assessments have been carried out in recent years to evaluate the Food Banks Program, aiming to verify the performance of the public management. In this regard: (i) the audit carried out by the Federal Court of Accounts [27] and its respective monitoring [28,29]; (ii) the first evaluation of the Food Banks Program, which mapped and characterized exclusively the governmental facilities, in 2006 [30]; (iii) the second evaluation of the Food Banks Program, which assessed the implementation and management of governmental units, in 2011 [31]; (iv) and the evaluation of the structure, processes, and results of government food banks in the Brazilian state of Minas Gerais, in 2014 [2]. All these mentioned technical-scientific studies, however, were restricted to the governmental food banks and to some territory, and a nationwide survey encompassing all types of food banks is missing.

González-Torre and Coque [11] and Simmet et al. [32] noted that the food banks received little attention in international literature. This is also true for the Brazilian scenario, which indicates the great need for studies and research that contribute to the cycle of agenda definition, formulation, decision-making, implementation, and evaluation of Brazilian food banks.

Motivated by this context, the aim of this article is to present an unprecedented overview of all the food banks currently in operation across the entire Brazilian territory, discussing their performances and perspectives regarding: (i) reducing food loss and waste; (ii) improving the food security of the beneficiaries and (iii) development of educational actions.

## 2. Materials and Methods

### 2.1. Sample Selection

This study encompassed all the Brazilian food banks distributed in the 27 federative units of the country, and we performed observational research, with a descriptive, quantitative, and exploratory approach, with a cross-sectional survey.

Until the time of this research, there was no mapping of all Brazilian food banks and, therefore, this unprecedented study had the challenge of identifying, locating, and characterizing the facilities distributed across the entire Brazilian territory. From January to July 2018, a survey of the universe of Brazilian food banks was carried out, with support from the Ministry of Citizenship and the National Department of Sesc which provided data from the Brazilian Food Bank Network and from Rede Mesa Brasil, respectively. To complement the mapping, an exhaustive active search was carried out for existing and operating facilities in the country through the researchers’ networks and internet news.

After this systematization, it was possible to identify 233 Brazilian food banks and include them in the sample universe of the research.

### 2.2. Data Collection Instrument

The development of the data collection instrument was guided by intense bibliographic research [2,33,34,35,36]. Tenuta [2] adapted assessment variables referring to the dimensions of structure, processes and results, originally used to assess the quality of health services [36], to the concepts of food banks. The food bank evaluation variables proposed by Tenuta [2] were adapted for this study, guiding the construction of the questions in a semi-structured questionnaire, which was evaluated by managers and technicians from six operating food banks, to test its validity and reliability. The instrument was then revised to incorporate the obtained contributions, generating the final version of the questionnaire with 69 questions. For this paper, 16 questions were selected and analyzed (questionnaire). The final version of the questionnaire was implemented for application using the Google Forms tool.

Besides the questions, an initial text was included in the instrument, presenting an overview of the research, its supporters, executors, and objectives, in addition to emphasizing the importance of participation. Other information on filling in, deadlines for participation, and contact of those responsible for the research were also added. Consent for participation was requested at the beginning of the questionnaire itself, using the answer options “YES” and “NO” for the following text: “Do you accept to participate in the “National Evaluation of Food Banks” research?” (Mandatory question). The link to access the full content of the free and informed consent form was included in the text.

The self-completed questionnaire was sent on 20 December 2018, via institutional email, to all food banks registered in the database of contacts systematized by the research team (*n* = 233).

Aiming at the adherence of those responsible for food banks, we kept constant telephone and email contact during the participation period, from 20 December 2018 to 15 March 2019. No incentive was offered to participate and complete the questionnaire.

### 2.3. Research Variables

The variables of interest extracted from Tenuta [2] and adapted by this study were grouped based on the three fundamental goals of the Brazilian food banks (Table S1). It is understood that an overview of the experiences of Brazilian food banks, the objective of this work, must be based on the guiding pillars of such social action.

### 2.4. Geographic Analysis and Descriptive Statistics

The spatial distribution of all food banks was registered, on a map, using the ArcGis^®^ software (version 10.1, ESRI, (Redlands, CA, USA)). The information on management modality was associated with the identification of each food bank on the map so that it was possible to identify the capillarity of each modality in the country.

Descriptive statistics using absolute and relative frequencies was used to reveal the characteristics of Brazilian food banks related to the above-mentioned variables of interest, and to present other practices that contribute to improving the performance of Brazilian food banks.

All subjects provided their informed consent for inclusion before they participated in the study, and all signed a free and informed consent form. The study was conducted in accordance with the Declaration of Helsinki, and the protocol was approved by the Ethics Committee of Federal University of Vales do Jequitinhonha e Mucuri (Diamantina, Brazil) (No 2.633.526, 3 May 2018).

## 3. Results

The research questionnaire was sent to the 233 food banks mapped in Brazil, obtaining a return and participation rate of 97.42% (227 food banks). Participation was voluntary, and six food banks did not answer the questionnaire for undeclared reasons. Out of the 227 respondent food banks, 10 (4.41%) were excluded from the sample group because they reported not being currently in operation, totaling 217 units analyzed in this study.

The results are presented below to characterize the Brazilian food banks and their distribution in the country. Later, the descriptive statistical results serve to draw an overview of the performance of this facility according to the variables of interest in this research, that is, the fundamental goals of Brazilian food banks.

### 3.1. Management and Operation: Who Are the Brazilian Food Banks

The management of food banks in Brazil occurs in four main modalities, described according to the typology defined by the Brazilian Food Bank Network [25]. Public food banks are those managed by the municipal governments. Most of them have support of the federal government, which since 2005 has financed the implementation of new units or the physical renovation of existing structures, but the operating and maintenance expenses are paid by the city halls. Public food banks represent 42.86% (*n* = 93) of the units operating in the country.

The other three management modalities are related to non-public initiatives (Table 1). Rede Mesa Brasil Sesc food banks are facilities implemented and maintained by the Social Service of Commerce (Sesc), a non-profit Brazilian private institution. The activities of the food banks are financed by regional departments existing in each state, often with financial support from the Sesc national department. This is the second most common management modality of the Brazilian food banks, accounting for 41.01% (*n* = 89) of the investigated units.

The food banks of civil society organizations are spontaneous civil society initiatives, which are implemented and maintained with resources from companies and other supporting partners, also receiving individual donations of money, services or products. These facilities often benefit from public or private notices for supporting social actions. These food banks represent 11.98% (*n* = 26) of the studied units.

Finally, there are the food banks located within supply centers, which are generally large commercial centers that concentrate dozens to hundreds of stores that sell food, both wholesale and retail. These food banks are financially maintained and managed by the supply centers themselves. The former MDS supported, in 2012, via a public notice, the implementation or modernization of these facilities, which are strategically located. This management modality is the least frequent, accounting for 4.15% (*n* = 9) of the Brazilian food banks.

**Figure 1:**
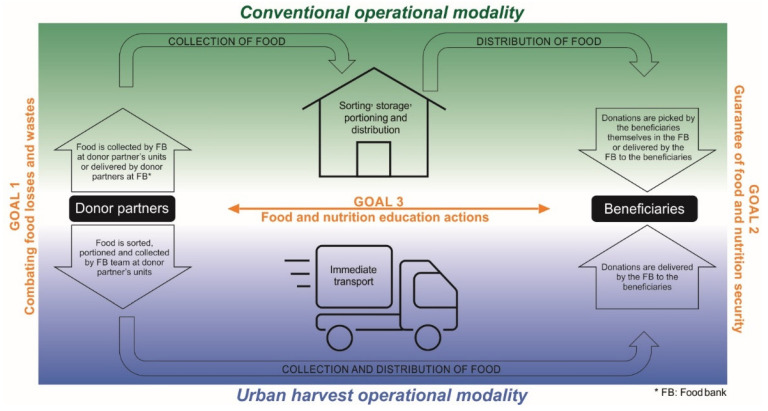
Operational logic of the Brazilian food banks and their relationship with the three fundamental goals.

**Figure 2:**
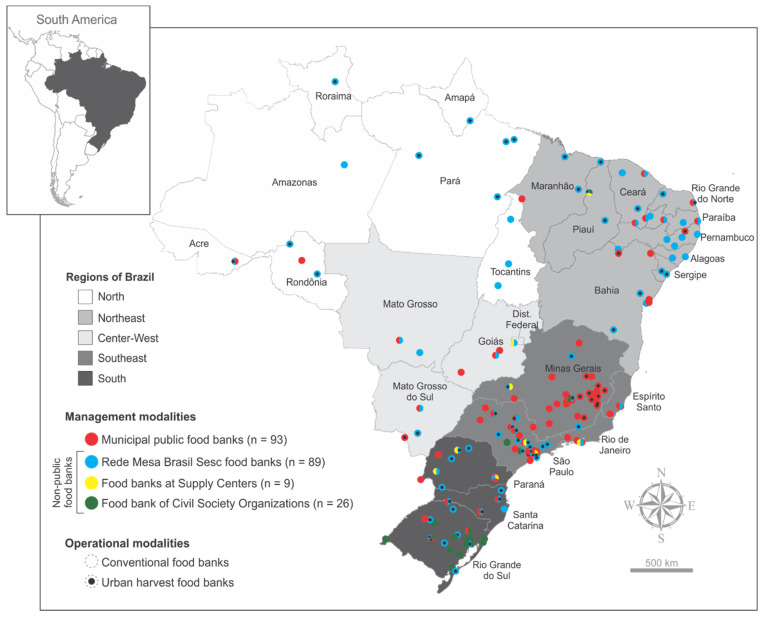
Distribution of all the food banks operating in Brazil in 2019, according to their management and operational modalities (*n* = 217).

In Brazil, food banks also present two different operational modalities (Figure 1 and Table S2). The first modality identified by this study is the one in which the food bank is headquartered in a building adapted for administrative and operational services such as the sorting, storage, portioning, and distribution of food for donation to beneficiaries. They may also have space and equipment for handling and processing food, producing sauces, pulps, and sliced vegetables, among other minimally processed foods. In this case, either the donor partners can deliver donations in the food bank facility, or the food bank vehicles may gather it from the donors’ facilities, and the beneficiaries themselves are responsible for withdrawing donations directly in the food bank. This operational modality is recognized in Brazil as a conventional modality and is referenced in this work for analysis purposes (Figure 1 and Figure 2). The second operational modality, on the other hand, is that in which only the administrative activities of the food bank are carried out in a headquarter. The operational team of such food banks goes to the donor partners to collect donation, and performs, in the donor establishment itself, the stages of sorting and portioning, followed by the immediate delivery of food to the institutions and/or beneficiary families. This modality is recognized in Brazil as an urban harvest modality and is referenced in this work for analysis purposes (Figure 1 and Figure 2). It was observed that, in Brazil most food banks (64.52%) operate in the conventional modality, and 35.48% in the urban harvest modality (Table S2). Analyzing by management modality, food banks located in supply centers operate only in the conventional modality (100.00%). Most food banks of Rede Mesa Brasil Sesc operate (66.30%) in the urban harvesting modality. The civil society organizations (92.30%) and public (82.80%) food banks, on the other hand, operate mostly in the conventional modality.

### 3.2. Geographic Analysis: Where Are the Brazilian Food Banks

There are identified food banks operating in the 27 Brazilian federative units, but they are heterogeneously distributed throughout country territory, being most concentrated in the southeast region, followed by the south and northeast. Figure 2 and Table 1 and Table S2 summarize the current spatial distribution of the food banks in operation in Brazil, according to their management and operational modalities, making it possible to visualize the capillarity of each type in the country.

Public food banks are most concentrated in the southeast region, highlighting the states of Minas Gerais (*n* = 36) and São Paulo (*n* = 20). Several states, especially from the north region, do not present public food banks in operation. The Rede Mesa Brasil Sesc food banks are the only ones present in all 27 federative units.

Food banks from civil society organizations are largely concentrated in the state of Rio Grande do Sul, which account for 20 of the 26 food banks in this modality. The other six are in São Paulo, Minas Gerais and Piauí states. Finally, the few supply centers food banks are mainly located in the Southern and South regions.

Most of the Brazilian food banks operate in the conventional operational modality. Although disseminated throughout the country, food banks operating in the urban harvest modalities are more concentrated in the southeast and south regions (Figure 2).

### 3.3. Overview of the Performance of Brazilian Food Banks According to Their Fundamental Goals

#### 3.3.1. Performance of Food Banks in Achieving Goal 1: Combating Food Losses and Waste

The profile of the donor partners of Brazilian food banks was investigated, aiming to trace the main provenance of the recovered food. The survey showed that 46.54% of the food banks (*n* = 101) operating in the country have warehouses, markets, supermarkets, and hypermarkets as their main donors, which indicates that they articulate preferably with donors at the end of the food supply chain. This characteristic, verified at a national level, differs only in the food banks of supply centers, which, due to their location, articulate preferably with donor partners installed in the center itself (Table 2).

Food banks can articulate with each other in networks to enhance food collections and improve logistics, among other actions. A total of 57.60% of the Brazilian food banks (*n* = 125) participate in local and/or regional food bank networks. Almost all the food banks of civil society organizations (92.31%) integrate some local and/or regional network, followed by public food banks (58.06%), food banks of the Rede Mesa Brasil Sesc (48.31%), and the food banks of supply centers (44.44%).

The results indicated that some alternative practices are frequently used by the Brazilian food banks to complement their operational inventories. In 2018, 50.69% (*n* = 110) of food banks reported that they bought food through the Food Acquisition Program (PAA), a public program for purchasing food from family farming. Of these, 29.09% (*n* = 32) reported that the PAA share in the inventories was 1 to 25% of the total volume collected in that year, 24.55% (*n* = 27) reported that this share was 26% to 50%, 27.27% (*n* = 30) reported participation with 51% to 75%, and in 19.09% (*n* = 21) of these food banks the PAA share represented 76% to 100% of the food inventory.

Observing this characteristic by operational modality, public food banks (64.52%) were the ones that most operationalized the PAA compared with other modalities. In 2018, 48.31% of the Rede Mesa Brasil Sesc facility had this complementary inventory, followed by the supply centers food banks (33.33%), and food banks from civil society organizations (15.38%).

#### 3.3.2. Performance of Food Banks in Achieving Goal 2: Guarantee of Food and Nutritional Security

The profile of the main beneficiaries of the Brazilian food banks was also surveyed, as a premise to understand the impacts of their activity. It was found that 41.47% (*n* = 90) of Brazilian food banks have families at social risk as the main beneficiaries, while 36.87% (*n* = 80) of the food banks serves mainly children, through the delivery of food donations to institutions of childcare. These two beneficiary profiles are the most expressive ones in all four food bank management modalities (Table 3).

The families at social risk benefit from the activities of food banks in three main forms: (i) in 61.29% (*n* = 133) of the food banks the food is donated to registered mediating institutions that pass it to the families. Specifically, this practice is adopted by 48.39% of the municipal public food banks, 67.42% of the units of the Rede Mesa Brasil Sesc, 92.31% of the food banks of civil society organizations, and 44.44% of the supply center facility; (ii) in the second form, adopted by 31.34% (*n* = 68) of the food banks, the food is donated directly to families previously selected and registered by the Social Assistance Reference Centers (CRAS), which are municipal public welfare agencies. A total of 69.89% of the public food banks, 2.25% of the units of the Rede Mesa Brasil Sesc, and 11.11% of the facility at the supply centers use this strategy to serve families; (iii) in the last form, the food is also donated directly to the families, but the selection and registration of them is performed by the food bank itself. Only 5.53% (*n* = 12) of the food banks adopt this practice, comprising 8.60% of the public food banks, 3.37% of the Rede Mesa Brasil Sesc facilities, and 11.11% of units installed in supply centers. A total of 13.82% of food banks (*n* = 30) combine two or three of these practices to assist families, and the other 86.18% (*n* = 187) adopt a single form. When analyzed by management modality, public food banks stand out for being the ones that most articulate with CRAS to plan and carry out the service. The Rede Mesa Brasil Sesc prioritizes service to families through the support of mediating institutions.

The food banks may receive a wide range of products, and the type of food subsequently donated to the beneficiaries has a direct impact on their food and nutritional security. To evaluate this issue, the nutritional profile of foods most received by the food banks was investigated. It was found that 85.25% (*n* = 185) of the Brazilian food banks have “fruits and vegetables (natural, chilled or frozen, dried, and dehydrated—without adding any other ingredients)” as the most recurrent food type in operational inventories. This characteristic maintains in 96.63% of the facility of the Rede Mesa Brasil Sesc, in 91.40% of the municipal public food banks, and in 88.89% of those installed in supply centers. The basic staples of the Brazilian diet, “rice, corn in grain or on the cob, beans, manioc, corn, and wheat flour, pasta, homemade bread, and French bread” [37], are the main received items in 13.82% (*n* = 30) of the food banks. The last food group is more recurrent in the inventory of 76.92% of civil society organizations food banks.

The quality of the food collected by the food banks is also a major factor to analyze, because it impacts directly in the volume of food that is suitable for consumption (and, therefore, donation) after selection. In total, 90.32% (n = 196) of the Brazilian food banks reported that they use up to 75% of the total volume of food collected. Of these, the food banks of civil society organizations and the Rede Mesa Brasil Sesc stand out for having the highest recovery rates, using 96.15% and 95.51% of the collected food, respectively. A total of 6.91% (*n* = 15) of the investigated food banks manage to use less than 50% of the collected volume, and these represent 22.22% of the supply centers food banks, 8.60% of the public food banks, 4.49% of the Rede Mesa Brasil Sesc food banks, and 3.85% of the food banks from civil society organizations. The remaining 2.77% food banks use up to 75% of the volume of food collected, the utilization range occupied by 6.45% of the municipal public food banks.

In terms of food volume, Brazilian food banks annually move thousands of kilos of food that, otherwise, would be lost or wasted throughout the supply chain. Most of the investigated food banks recover and donate over 20 tons of food annually, and some of them go far beyond that mark. The analysis of the food volume donated by food banks of each management modality highlights the huge operational capacity of the food banks located in supply centers. In 2018, 66.67% of such food banks donated more than 450 tons of food to their beneficiaries (Table 4).

#### 3.3.3. Performance of Food Banks in Achieving Goal 3: Performing Food and Nutrition Education Actions

Brazilian food banks have the actions of food and nutrition education as a strategic agenda, which can target the audience of donor partners, the food bank collaborators and employees, or the beneficiaries. Educational actions are carried out by food banks more frequently (87.56%, *n* = 190) for institutions, beneficiary families and individuals, followed by actions aimed at their collaborators and employees (86.64%, *n* = 188). Educational activities are offered to donor partners by 75.12% (*n* = 163) of food banks. The food banks of the Rede Mesa Brasil Sesc and the supply centers food banks are the ones that most prioritize educational actions aimed at donor partners and institutions, beneficiary families and individuals (Table 5).

### 3.4. Practices That Contribute to Improving the Performance of Brazilian Food Banks

Evaluation and monitoring practices, accountability, satisfaction surveys, and nutritional assessment of beneficiaries are actions performed by the food banks that substantially impact the efficiency of daily operational tasks and the fulfillment of the three fundamental goals. A total of 73.27% (*n* = 159) of the Brazilian food banks report their results in activities/events with partners, beneficiaries, and other members of the civil society. Self-assessment (65.90%, *n* = 143) and the dissemination of results in the media (58.99%, *n* = 128) are also recurrent practices among Brazilian food banks. Satisfaction surveys with beneficiary institutions (29.03%, *n* = 63), with their employees and collaborators (23.50%, *n* = 51), and monitoring the nutritional status of beneficiaries (23.04%, *n* = 50) are also actions carried out by about one-third of the food banks surveyed. Satisfaction surveys with donor partners, is a practice performed by few facilities (17.51%, *n* = 38) (Table 6).

## 4. Discussion

This study revealed the presence of food banks operating in all the 27 Brazilian federative units, indicating the existence of a comprehensive network of food banks in Brazil. southeast, south, and northeast are the most populous regions of the country and, coherently, are the regions with more food banks. It is noteworthy, however, that there are large regions of the country without food banks, and that some major urban centers, such as Florianópolis, Manaus and Belém, for example, are assisted by only one food bank.

The public food bank is the most common management modality of the country, followed by the Rede Mesa Brasil Sesc modality. However, it should be noted that Rede Mesa Brasil Sesc is the only food bank management modality present in all Brazilian states. The remarkable capillarity of these two management modalities is due to the constant financial investments made by the federal government (mainly through public notices between 2005 and 2012) to support the implantation and modernization of public food banks throughout the country and, in the same way, the important financial investment that Sesc made in their food bank system in recent years.

We found that Brazilian food banks operate in two distinct routes, implementing a physical structure for handling and storing food, or not. In other countries, the operation of food banks is usually similar to the conventional modality, described as the most recurrent in Brazil [11,16]. This operational dynamic allows greater flexibility in the process and enhances the use of the collected food since it is possible to have its own and exclusive physical area for sorting and, above all, more time for the processes. On the other hand, the urban harvesting modality is characterized by more agile actions, requiring less physical and financial resources to implement and operate. The choice for an operational modality must consider what is available to make the facility operate, besides assessing the objectives and goals outlined for the activity.

There are some relevant relations between operational and management modalities. Given the logistical conditions and potential for handling large volumes of food, the food banks located in supply centers choose, in their entirety, to have a physical structure available for sorting and storing food, operating in the conventional modality. The Rede Mesa Brasil Sesc, in turn, is the only management modality in which most of its units operate in the urban harvest modality. The choice for an operational modality, in the Rede Mesa Brasil Sesc, considers the relationship between cost and benefit regarding human resources and financial investments necessary to operate, depending on the flow of donations, availability of donor partners and needs of beneficiary institutions [38].

Another important relation revealed by the data is that Brazilian food banks have been primarily collecting food from donor partners at the final stage of the supply chain (such as warehouses and markets), which is likely related to the location of such partners in urban areas, which greatly facilitates logistics due to the road network. In the two food bank evaluation surveys, carried out in 2006 and 2011, the supply centers were identified as the major donor partners of the food banks [30,31], which indicates a substantial change in the food sources in the last decade. Understanding where the food banks are gathering most of their food is a crucial step to identify the stages of the production and supply chain that are not addressed by the current activities, and trace future strategies. According to data from the Food Losses and Waste in Latin America and The Caribbean report [4], 28% of food losses occur in the production segment, 6% during processing, 22% in handling and storage, 17% during marketing and distribution and 28% in the consumer segment. Therefore, the results of this study indicate the need for Brazilian food banks to expand food collection in the sectors of food production, processing, handling and storage, where the most significant losses occur. In this sense, the networked articulations verified by this study become a concrete possibility for strategic interaction aiming to mobilize potential donor partners belonging to other stages of the production and supply chain.

Analyzing the composition of the operating inventories of the studied facilities allowed us to diagnose an important pattern in the food banks performances. The purchase of food from family farming with government resources, through PAA, was verified in half of the Brazilian food banks, but especially in the public ones, corroborating a practice identified by the second food bank evaluation survey [31] and by Tenuta and Teixeira [39]. Such a pattern deserves attention, because as the food purchases grow in importance within the food banks, these units deviate from their primary objective of combating food losses and wastes. Taking this issue into account, the Brazilian Food Bank Network stated that, in order to be considered a food bank and integrate the network, a unit must necessarily collect food that was about to be lost and/or wasted, and these products must compose at least 25% of their operational inventories. Therefore, to be considered a food bank, a unit must not exceed 75% of food coming from other programs, such as PAA [40]. Using this parameter, according to their 2018 data, 19.09% of the studied units would not be considered food banks and should be classified as warehouses for family farming products instead. However, as this parameter is dynamic, it was decided not to exclude these units from the sample so that it was possible to bring this question into the analysis. The first audit of public food banks carried out by the Federal Court of Accounts in 2005, already indicated that part of the audited facilities worked almost exclusively with food that did not originate from losses and waste, constituting a distortion of one of the fundamental goals of food banks [27].

In line with the objective of guaranteeing food and nutritional security, Brazilian food banks have maintained their concern with encouraging the consumption of healthy foods. We verified the predominance of donations of natural fruits and vegetables to food bank beneficiaries, a pattern already identified in the two Food Bank Evaluation Surveys [30,31]. Food banks have served primarily families at social risk, with fruits, vegetables, and other items of the traditional Brazilian diet. According to the Food Guide for the Brazilian Population [41], a healthy diet must be composed of natural or minimally processed foods, in great variety and predominantly of vegetable origin. In this sense, we consider that the Brazilian food banks have been moving toward the promotion of adequate and healthy food. This concern with nutritional quality of the food offered by the food banks is legitimate due to recent evaluation of the profiles of food consumption of the Brazilian population, which reported a tendency toward the consumption of ultra-processed food in detriment to the consumption of healthy foods [42].

The systematic promotion of food and nutritional education activities is an innovative feature of Brazilian food banks. The results of our research demonstrate a constant concern of the food banks in carrying out such educational actions, in continuity with the report by the First Food Bank Evaluation Survey [30]. In this regard, the Brazilian Food and Nutrition Education Benchmark for Public Policies recognizes the food banks as important spaces for promoting and strengthening food and nutrition education [43]. The great potential of food banks to promote the practice of healthy eating habits to a wide and diverse audience, from donor partners, collaborators, and employees to beneficiary institutions, families and individuals is noteworthy.

Another important result of this survey was the description of common practices such as self-assessment and public presentations of results among the Brazilian food banks. Disclosure of results and communication are crucial elements to increase the social visibility of the goals and activities of the food banks, contributing to the establishment of new partnerships and to the consolidation of their actions. Self-assessment, in turn, enables greater efficiency in the allocation of physical, human, and financial resources and increases the autonomy and responsibility of the technical and administration teams. According to our research, satisfaction surveys have not been common practice in the Brazilian food banks. Such practices should be incorporated into the routine of the food banks to evaluate the satisfaction of the beneficiaries about the provided services and about the quality of the donated food, as well as to verify the efficiency of the processes involving donor partners.

## 5. Conclusions

In an antagonistic world scenario where one-third of the food produced for human consumption is lost or wasted, millions of people suffer from hunger and/or are in some degree of food insecurity [8]. Global commitments, such as goals 2.1 and 12.3 of the United Nations Sustainable Development Goals [44], are being systematically assumed by various countries to change this reality. In this context, food banks all around the world, including in Brazil, have a strategic role in reintroducing lost or wasted food in the production and supply chain to supplement food for people in situations of social, food, and nutritional vulnerability.

This study revealed the great reach of the food bank strategy in the Brazilian territory, figured as a comprehensive and widespread network that spread throughout the country. The irregular spatial distribution of the units, however, reveals regions that have little equipment in place for operating, indicating the need for the expansion of services. The public authorities and the Rede Mesa Brasil Sesc have, in recent years, been the main motivating force for implementing and supporting the food banks in Brazil. However, this study points to the need and relevance of new initiatives located in or close to supply centers given the percentage of loss occurring in the distribution phase of the supply chain, which reveals the potential, and responsibility, of rescuing large volumes of food in these spaces.

Brazilian food banks relied on international experiences to shape their performance, but, over time, they have developed their unique modus operandi, and adapted to the country’s social, territorial, political, and economic reality. In Brazil, two operational modalities forge the logistical format of food banks. The capturing, handling, and distribution of food donations are carried out under two different perspectives that balance financial investments and the infrastructure required to fulfill the agenda of reducing food losses and wastes and guaranteeing the human right to adequate food.

When combating food loss and waste, the Brazilian food banks have demonstrated commitment in attracting food donations from partners at the marketing stage of the supply chain, but have left unattended the stages of the production, transportation, processing, and distribution of food. This behavior reveals the need for new fronts of action for the Brazilian food banks which allow, in addition to increasing the collected volumes, to intensify the rescue of food that is about to be lost. It is imperative that the food banks’ teams themselves systematically analyze the need to attract new donor partners and, to that end, continue mapping new potential partners in their coverage area.

It is also worth questioning the composition of the operational inventories of food banks. Previous studies identified, and the present research corroborates, that the Brazilian food banks have been purchasing food to complement their inventories, indicating a potential distortion of the primary objective of collecting food from losses and waste.

The objective of guaranteeing food and nutritional security for vulnerable populations reinforces and justifies the place occupied by food banks on the public agenda and the support received by other private, parastatal, and civil society institutions and organizations. The current concept of food and nutritional security considers the food dimension and the nutritional dimension as basic and complementary elements for the manifestation of the physical availability and nutritional quality of food. This reflection must be intrinsic to the performance of food banks, and our study indicates that Brazilian facilities are succeeding in offering healthy food to beneficiaries, meeting the recommendations for adequate health and lifestyle habits.

Food and nutrition education is an essential dimension to guarantee the human right to food, because it can significantly guide the eating behavior of individuals, from purchasing to consuming food. Our research identified that Brazilian food banks have engaged in such educational actions, spreading information and encouraging the appropriation of knowledge regarding healthy food habits to donor partners, collaborators, employees, and beneficiaries. However, it should be noted that information on the contents and frequencies of such food and nutrition education actions were not assessed by this research, pointing to the need for new studies capable of understanding the nature, scope, and pedagogical processes developed in educational actions offered by food banks, besides assessing the impact of these actions for the audience involved and participating.

This study has limitations for comparison and discussion with previous research because it is the first survey involving Brazilian food banks from the entire national territory analyzing all existing management and operational modalities. We find extremely important that more studies with this wide scope are carried out in the future, aiming for the constant improvement of the food banks. We hope that the results, discussions, and perspectives presented in this study serve as a subsidy to recognize, improve, and strengthen Brazilian food banks and other food banks in operation around the world.

## Figures and Tables

**Table 1:** Distribution of food banks in Brazil, by region and by state, in 2019 (*n* = 217).

	Municipal Public Food Banks		Non-Public Food Banks † (Rede Mesa Brasil Sesc, CSOs *, Ceasas **)		Total
**BRAZILIAN STATES**	**93**	**42.86%**	**124**	**57.14%**	**217**
**NORTH REGION**	**2**	**13.33%**	**13**	**86.67%**	**15**
Amazonas	0	0.00%	1	100.00%	1
Acre	1	50.00%	1	50.00%	2
Rondônia	1	33.33%	2	66.67%	3
Roraima	0	0.00%	1	100.00%	1
Amapá	0	0.00%	1	100.00%	1
Pará	0	0.00%	4	100.00%	4
Tocantins	0	0.00%	3	100.00%	3
**NORTHEAST REGION**	**12**	**28.57%**	**30**	**71.43%**	**42**
Maranhão	1	33.33%	2	66.67%	3
Piauí	0	0.00%	5	100.00%	5
Rio Grande do Norte	1	33.33%	2	66.67%	3
Ceará	2	33.33%	4	66.67%	6
Paraíba	3	37.50%	5	62.50%	8
Bahia	4	57.14%	3	42.86%	7
Pernambuco	1	16.67%	5	83.33%	6
Alagoas	0	0.00%	2	100.00%	2
Sergipe	0	0.00%	2	100.00%	2
**SOUTHEAST REGION**	**63**	**65.63%**	**33**	**34.38%**	**96**
Minas Gerais	36	85.71%	6	14.29%	42
Espírito Santo	3	75.00%	1	25.00%	4
Rio de Janeiro	4	66.67%	2	33.33%	6
São Paulo	20	45.45%	24	54.55%	44
**SOUTH REGION**	**10**	**19.61%**	**41**	**80.39%**	**51**
Santa Catarina	3	37.50%	5	62.50%	8
Paraná	4	30.77%	9	69.23%	13
Rio Grande do Sul	3	10.00%	27	90.00%	30
1/2					
2/2					
**CENTRAL-WEST REGION**	**6**	**46.15%**	**7**	**53.85%**	**13**
Goiás	3	75.00%	1	25.00%	4
Mato Grosso	1	33.33%	2	66.67%	3
Mato Grosso do Sul	2	50.00%	2	50.00%	4
Federal District	0	0.00%	2	100.00%	2

† The number of non-public food banks, presented by the management method of Rede Mesa Brasil Sesc, civil society organizations (CSOs), and Ceasas, by region and state, are presented in a spreadsheet attached to this manuscript; * food banks of civil society organizations; ** food banks located in supply centers.

**Table 2:** Distribution of Brazilian food banks, according to donor partners and management modality, in 2019 (*n* = 217).

Donor Partners	Management Modalities of Food Banks			
	Municipal Public Food Banks (*n* = 93)	Rede Mesa Brasil Sesc Food Banks (*n* = 89)	Food Banks of Civil Society Organizations (*n* = 26)	Food Banks Located in Supply Centers (*n* = 9)
Warehouses, markets, supermarkets, hypermarkets	31.18%	53.93%	88.46%	0.00%
Supply center	10.75%	15.73%	7.69%	55.56%
Family farming	31.18%	6.74%	0.00%	11.11%
Medium and large-scale agriculture	1.08%	16.85%	0.00%	11.11%
Solidarity campaigns	10.75%	0.00%	0.00%	11.11%
Other food banks	5.38%	0.00%	0.00%	0.00%
Other origins *	9.68%	6.74%	3.85%	11.11%

* Food industries, apprehension and traffic accidents, individuals.

**Table 3:** Main beneficiaries of the Brazilian food banks, according to management modality, in 2019 (*n* = 217).

Beneficiaries	Management Modalities of Food Banks			
	Municipal Public Food Banks (*n* = 93)	Rede Mesa Brasil Sesc Food Banks (*n* = 89)	Food Banks of Civil Society Organizations (*n* = 26)	Food Banks Located in Supply Centers (*n* = 9)
Families at social risk	51.61%	23.60%	76.92%	11.11%
Children	24.73%	57.30%	7.69%	44.44%
Adults	6.45%	6.74%	7.69%	11.11%
Adolescents	3.23%	8.99%	0.00%	0.00%
Specific populations *	4.30%	0.00%	3.85%	0.00%
Older people	2.15%	1.12%	3.85%	0.00%
Varied audience **	7.53%	2.25%	0.00%	33.33%

* Homeless population and people with chemical dependence; ** when there was no specification of the age group or special condition of the beneficiary audience.

**Table 4:** Volume of food donated by Brazilian food banks to beneficiary institutions and families by management modalities in 2018 (*n* = 217).

Tons of Donated Food	Management Modalities of Food Banks			
	Municipal Public Food Banks (*n* = 93)	Rede Mesa Brasil Sesc Food Banks (*n* = 89)	Food Banks of Civil Society Organizations (*n* = 26)	Food Banks Located in Supply Centers (*n* = 9)
0 to 1 ton *	16.13%	1.12%	0.00%	11.11%
1 to 20 tons	32.26%	1.12%	38.46%	0.00%
21 to 70 tons	20.43%	5.62%	34.62%	22.22%
71 to 150 tons	10.75%	22.47%	7.69%	0.00%
151 to 300 tons	10.75%	26.97%	11.54%	0.00%
301 to 450 tons	6.45%	14.61%	0.00%	0.00%
More than 450 tons	3.23%	28.09%	7.69%	66.67%

* 1 ton = 1000 kg.

**Table 5:** Types of beneficiaries attended by Brazilian food banks in 2019 (*n* = 217).

Audiences		Management Modalities of Food Banks			
		Municipal Public Food Banks (*n* = 93)	Rede Mesa Brasil Sesc Food Banks (*n* = 89)	Food Banks of Civil Society Organizations (*n* = 26)	Food Banks Located in Supply Centers (*n* = 9)
Donor partners	Yes	47.31%	98.88%	92.31%	77.78%
	No	52.69%	1.12%	7.69%	22.22%
Collaborators and employees	Yes	78.49%	92.13%	100.00%	77.78%
	No	21.51%	7.87%	0.00%	22.22%
Beneficiary institutions, families, and individuals	Yes	73.12%	100.00%	96.15%	88.89%
	No	26.88%	0.00%	3.85%	11.11%

**Table 6:** Practices that contribute to improving the performance of Brazilian food banks in 2019 (*n* = 217).

Practices	Management Modalities of Food Banks			
	Municipal Public Food Banks (*n* = 93)	Rede Mesa Brasil Sesc Food Banks (*n* = 89)	Food Banks of Civil Society Organizations (*n* = 26)	Food Banks Located in Supply Centers (*n* = 9)
(**a**)	56.99%	64.04%	100.00%	77.78%
(**b**)	50.54%	57.30%	92.31%	66.67%
(**c**)	51.61%	93.26%	92.31%	44.44%
(**d**)	22.58%	34.83%	38.46%	11.11%
(**e**)	8.60%	21.35%	38.46%	11.11%
(**f**)	21.51%	20.22%	46.15%	11.11%
(**g**)	25.81%	15.73%	38.46%	22.22%
(**h**)	11.83%	1.12%	0.00%	0.00%

(**a**) Self-assessment; (**b**) dissemination of their results in the media; (**c**) dissemination of their results in activities/events with partners and beneficiary institutions; (**d**) satisfaction survey with beneficiary institutions; (**e**) satisfaction survey with donor partners; (**f**) satisfaction survey with their employees and collaborators; (**g**) monitoring the nutritional status of users of institutions and their families; (**h**) does not perform any of the practices.

## Data Availability

The data presented in this study are available on request from the corresponding author. The data are not publicly available due to agreements with the funding institution.

## Supplementary Materials

The following are available online at https://www.mdpi.com/article/10.3390/ijerph182312598/s1, Table S1: Goals of Brazilian food banks and research variables, Table S2: Distribution of food banks in Brazil in 2019, by region and by state, according to operational modality (*n* = 217).

## References

[1] Riches G. (2002). Food Banks and Food Security: Welfare Reform, Human Rights and Social Policy. Lessons from Canada?. Soc. Policy Adm..

[2] Tenuta N. (2014). Análise tridimensional da situação dos Bancos de Alimentos de Minas Gerais, Brasil. Master’s Thesis.

[3] Henz G.P., Porpino G. (2017). Food losses and waste: How Brazil is facing this global challenge?. Hortic. Bras..

[4] FAO (2016). Food Losses and Waste in Latin America and The Caribbean. Newsletter 3. http://www.fao.org/3/i5504e/i5504e.pdf.

[5] Gustavsson J., Cederberg C., Sonesson U., van Otterdijk R., Meybeck A. (2011). Global food losses and food waste. Rome: Food and Agriculture Organization of the United Nations. http://www.fao.org/docrep/014/mb060e/mb060e00.pdf.

[6] Belik W.B., Cunha A.R.A.A., Costa L.A. (2012). Crise dos alimentos e estratégias para a redução do desperdício no contexto de uma política de segurança alimentar e nutricional no Brasil. Planej. Políticas Públicas.

[7] FAO Rome Declaration on World Food Security and World Food Summit Plan of Action. Proceedings of the World Food Summit.

[8] FAO, IFAD, UNICEF, WFP, WHO (2019). The State of Food Security and Nutrition in the World 2019. Safeguarding against Economic Slowdowns and Downturns. http://www.fao.org/3/ca5162en/ca5162en.pdf.

[9] Middleton G., Mehta K., McNaughton D., Booth S. (2018). The experiences and perceptions of food banks amongst users in high-income countries: An international scoping review. Appetite.

[10] Feeding America Our History. https://www.feedingamerica.org/about-us/our-history.

[11] González-Torre P.L., Coque J. (2016). How is a food bank managed? Different profiles in Spain. Agric. Hum. Values.

[12] The Global Foodbanking Netwoork Our Global Reach. https://www.foodbanking.org/what-we-do/our-global-reach/.

[13] Curtis K.A., Mcclellan S. (1995). Falling through the safety net: Poverty, food assistance and shopping constraints in an American city. Urban Anthropology and Studies of Cultural Systems and World Economic Development.

[14] Tarasuk V., Dachner N., Hamelin A.-M., Ostry A., Williams P., Bosckei E., Poland B., Raine K. (2014). A survey of food bank operations in five Canadian cities. BMC Public Heal..

[15] Li Y., Zhao W., Zheng H., Zhao F. (2014). A Study of Food Bank Impact on China\’s Charity and Food Security System. Adv. J. Food Sci. Technol..

[16] Gharehyakheh A., Sadeghiamirshahidi N., Ng B., Nepal E., Keathley S.E.H. (2018). A Sustainable Approach in Food Bank Logistics. Proceedings of the International Annual Conference of the Anais da American Society for Engineering Management, Coeur d’Alene, ID, USA, 17–20 October 2018.

[17] Belik W. Politicas de Seguridad Alimentaria para las Areas Urbanas. Politicas de Seguridad Alimentaria y Nutrición em America Latina.

[18] ONG Banco de Alimentos (2018). Relatório de Atividades. https://bancodealimentos.org.br/wp-content/uploads/2021/04/OBA-relatorio-de-atividades-2018.pdf.

[19] Sesc (2014). 10 anos de Mesa Brasil Sesc Goiás. Sesc: Goiás, Brasil. https://www.sescgo.com.br/area-de-atuacao/assistencia/mesa-brasil.

[20] Rede de Bancos de Alimentos do Rio Grande do Sul Nossa História. http://www.redebancodealimentos.org.br/Pagina/179/Nossa-Historia.

[21] Brasil. Ministério do Desenvolvimento Social e Combate à Fome Manual de Implantação do Banco de Alimentos. 2006. Bra-sília, DF. http://www.mds.gov.br/backup/programas/seguranca-alimentar-e-nutricionalsan/banco-deealimentos/MANUAL%20DE%20IMPLANTAcaO%20DO%20BANCO%20DE%20ALIMENTOS.doc>.

[22] Brasil (2011). Seleção Pública de Propostas Para Apoio à Implantação ou Modernização de Bancos de Alimentos. Edital MDS/SESAN Nº 02/2011.

[23] Brasil (2012). Seleção Pública de Propostas Para Apoio à Implantação ou Modernização de Bancos de Alimentos em Centrais de Abastecimento. Edital MDS/SESAN Nº 05/2012.

[24] Brasil Decreto nº 7.272, de 25 de agosto de 2010. Regulamenta a Lei nº 11.346, de 15 de setembro de 2006, que cria o Sistema Nacional de Segurança Alimentar e Nutricional—SISAN com vistas a assegurar o direito humano à alimentação adequada, institui a Política Nacional de Segurança Alimentar e Nutricional–PNSAN, estabelece os parâmetros para a elaboração do Plano Nacional de Segurança Alimentar e Nutricional, e dá outras providências. Diário Oficial (da República Federativa do Brasil). Brasília: Casa Civil. Subchefia de Recursos Jurídicos. http://www.planalto.gov.br/ccivil_03/_Ato2007-2010/2010/Decreto/D7272.htm#:~:text=1o%20Este%20Decreto%20define,os%20par%C3%A2metros%20para%20a%20elabora%C3%A7%C3%A3o.

[25] Brasil (2016). Portaria nº 17, de 14 de Abril de 2016. Institui a Rede Brasileira de Bancos de Alimentos.

[26] Brasil Avaliação de Políticas Públicas: Reflexões acadêmicas sobre o desenvolvimento social e o combate à fome, v.1: Intro-dução e temas transversais. 2014. Brasília, DF: MDS. https://aplicacoes.mds.gov.br/sagirmps/ferramentas/docs/1%20Introdu%C3%A7%C3%A3o%20e%20Temas%20Transversais.pdf.

[27] TCU (2005). Relatório de Avaliação de Programa: Programa Banco de Alimentos.

[28] TCU (2006). Relatório de monitoramento de auditoria: Programa Banco de Alimentos.

[29] TCU (2008). Relatório de monitoramento: Programa Banco de Alimentos.

[30] Redes (2006). Pesquisa de Avaliação do Programa Bancos de Alimentos. Sumário Executivo.

[31] FEC, DATAUFF (2011). Pesquisa de Avaliação do Programa Banco de Alimentos—Segunda Avaliação.

[32] Simmet A., Tinnemann P., Stroebele-Benschop N. (2018). The German Food Bank System and Its Users—A Cross-Sectional Study. Int. J. Environ. Res. Public Health.

[33] Brousselle A. (2011). Avaliação: Conceitos e Métodos.

[34] Jannuzzi P.M. (2016). Monitoramento e Avaliação de Programas Sociais: Uma Introdução Aos Conceitos e Técnicas.

[35] Brasil (2007). Metodologias e Instrumentos de Pesquisas de Avaliação de Programas do MDS: Bolsa Família, Assistência Social, Segurança Alimentar e Nutricional./Romulo Paes-Sousa.

[36] Donabedian A. (1980). Basic approaches to assessment: Structure, process and outcome. Explorations in Quality Assessment and Monitoring.

[37] Marchioni D.M., Claro R., Levy R.B., Monteiro A.C. (2011). Patterns of food acquisition in Brazilian households and associated factors: A population-based survey. Public Heal. Nutr..

[38] Sesc (2017). Guia do Programa Mesa Brasil Sesc.

[39] Tenuta N., Teixeira R.A. (2017). A eficácia dos Bancos de Alimentos de Minas Gerais no combate às perdas e desperdícios de alimentos. Segurança Aliment. Nutr..

[40] Brasil Instrução Normativa nº 01, de 15 de maio de 2017. Dispõe sobre a adesão dos Bancos de Alimentos à Rede Brasileira de Bancos de Alimentos. Diário Oficial (da República Federativa do Brasil). Brasília: Ministério do Desenvolvimento Social e Agrário. Secretaria Nacional de Segurança Alimentar e Nutricional. https://www.in.gov.br/web/guest/materia/-/asset_publisher/Kujrw0TZC2Mb/content/id/19090747/do1-2017-06-01-instrucao-normativa-n-1-de-15-de-maio-de-2017-19090713.

[41] Brasil (2014). Ministério da Saúde. Secretaria de Atenção à Saúde. Departamento de Atenção Básica. Guia Alimentar Para a Popu-Lação Brasileira.

[42] Martins A.P.B., Levy R.B., Claro R., Moubarac J.C., Monteiro C.A. (2013). Participacao crescente de produtos ultraprocessados na dieta brasileira (1987–2009). Rev. Saúde Pública.

[43] Brasil. Ministério do Desenvolvimento Social e Combate à Fome (2012). Marco de Referência de Educação Alimentar e Nutricional Para as Políticas Públicas.

[44] UN (2015). Transformando Nosso Mundo: A Agenda 2030 para o Desenvolvimento Sustentável. Organização das Nações Unidas. ONUBR. https://nacoesunidas.org/pos2015/agenda2030/.

