# Supplementary material for: Brazilian Food Banks: Overview and Perspectives
Source: Int J Environ Res Public Health. 2021 Nov 29;18(23):12598. doi: 10.3390/ijerph182312598 (PMC8657059; doi:10.3390/ijerph182312598)
Supplement: Supplementary file 1 [file ijerph-18-12598-s001.zip › ijerph-1326445-supplementary.pdf]

International Journal of
Environmental Research and Public Health

MDPI

Supplementary Material

Table S1. Goals of Brazilian food banks and research variables.

Goals of Brazilian food banks	Research variables [a]
1. Combating food losses and wastes	Profile of partner donors<br>Network articulation
2. Guarantee of food and nutrition security	Beneficiary profile<br>Volume of food traded<br>Nutritional profile of traded foods
3. Food and nutrition education actions	Realization and profile of educational activities

[a] Evaluation variables extracted from Tenuta [2] and adapted by the authors.

Table S2. Distribution of food banks in Brazil in 2019, by region and by state, according to operational modality ($n = 217$).

	**Conventional food bank (n = 140)**		**Urban harvesting food banks (n = 77)**	
**BRAZIL**	**140**	**64.52%**	**77**	**35.48%**
**NORTH REGION**	**6**	**40.00%**	**9**	**60.00%**
Amazonas	1	100.00%	0	0.00%
Acre	1	50.00%	1	50.00%
Rondônia	0	0.00%	2	100.00%
Roraima	1	50.00%	1	50.00%
Amapá	0	0.00%	1	100.00%
Pará	0	0.00%	4	100.00%
Tocantins	3	100.00%	0	0.00%
**NORTHEAST REGION**	**28**	**66.67%**	**14**	**33.33%**
Maranhão	1	33.33%	2	66.67%
Piauí	2	40.00%	3	60.00%
Rio Grande do Norte	1	33.33%	2	66.67%
Ceará	5	83.33%	1	16.67%
Paraíba	8	100.00%	0	0.00%
Bahia	4	57.14%	3	42.86%
Pernambuco	5	83.33%	1	16.67%
Alagoas	2	100.00%	0	0.00%
Sergipe	0	0.00%	2	100.00%
**SOUTHEAST REGION**	**60**	**62.50%**	**36**	**37.50%**
Minas Gerais	28	66.67%	14	33.33%
Espírito Santo	4	100.00%	0	0.00%
Rio de Janeiro	6	100.00%	0	0.00%
São Paulo	22	50.00%	22	50.00%
**SOUTH REGION**	**35**	**68.63%**	**16**	**31.37%**
Santa Catarina	4	50.00%	4	50.00%
Paraná	9	69.23%	4	30.77%
Rio Grande do Sul	22	73.33%	8	26.67%

**CENTRAL-WEST REGION**	**11**	**84.62%**	**2**	**15.38%**
Goiás	4	100.00%	0	0.00%
Mato Grosso	4	100.00%	0	0.00%
Mato Grosso do Sul	1	33.33%	2	66.67%
Federal District	2	100.00%	0	0.00%
